# Supplementary material for: Salivary Protein Cyclin-Dependent Kinase-like from Grain Aphid Sitobion avenae Suppresses Wheat Defense Response and Enhances Aphid Adaptation
Source: Int J Mol Sci. 2024 Apr 23;25(9):4579. doi: 10.3390/ijms25094579 (PMC11083452; doi:10.3390/ijms25094579)
Supplement: Supplementary file 1 [file ijms-25-04579-s001.zip › ijms-2927021-supplementary.pdf]

Table S1 Primers used in this study

Primers	Forward primer	Reverse primer	Use of primers
SaCDK	ATGAATAAGTACAGGGAGTTGGG	TCATGGAAATATATTTACTTGGTATACACA	cDNA cloning
SaCDK-sacI-xbaI	cgagctcATGAATAAGTACAGGGAGTTGGG	gctctagaTCATGGAAATATATTTACTTGGTATACACA	
attB-SaCDK	ggggacaagtttgtacaaaaaagcaggcttcATGAATAAGTACAGGGAGTTGGG	ggggaccactttgtacaagaaagctgggtcTCATGGAAATATATTTACTTGGTATACACA	Gateway cloning
QSaCDK	AGATACTGCGAGATGATGGT	ATGGTGCCTGAATGCTTC	RT-qPCR
PR1	AGTGCAAGTCCACCCTCATC	CTTGGCCTTGGTGATCTCAT	
PAL	CCACCCTGGACAGATTGAA	ATGAGCGGGTTGTCGTTG	
FAD	TCCCATTCCACCTACTGC	GGACTCACCAATCCGAGA	
LOX	GACCAGCGAAACAACAACC	GCATACAATAGCGGGAACAC	
ß-actin	GGAAAATCAGTCTCGGTTCAG	TCATACAGCAGGCAAGCAC	
NADH	TTAACGGTATAGATGATCAGCA	CGAGGAGAACATGCTCTTAGAC	
